# Spontaneous Sleep-Like Brain State Alternations and Breathing Characteristics in Urethane Anesthetized Mice

**DOI:** 10.1371/journal.pone.0070411

**Published:** 2013-07-30

**Authors:** Silvia Pagliardini, Simon Gosgnach, Clayton T. Dickson

**Affiliations:** 1 Department of Physiology, University of Alberta, Edmonton, Alberta, Canada; 2 Centre for Neuroscience, University of Alberta, Edmonton, Alberta, Canada; 3 Women and Children’s Health Research Institute, University of Alberta, Edmonton, Alberta, Canada; 4 Department of Psychology, University of Alberta, Edmonton, Alberta, Canada; Hospital Nacional de Parapléjicos, Spain

## Abstract

Brain state alternations resembling those of sleep spontaneously occur in rats under urethane anesthesia and they are closely linked with sleep-like respiratory changes. Although rats are a common model for both sleep and respiratory physiology, we sought to determine if similar brain state and respiratory changes occur in mice under urethane. We made local field potential recordings from the hippocampus and measured respiratory activity by means of EMG recordings in intercostal, genioglossus, and abdominal muscles. Similar to results in adult rats, urethane anesthetized mice displayed quasi-periodic spontaneous forebrain state alternations between deactivated patterns resembling slow wave sleep (SWS) and activated patterns resembling rapid eye movement (REM) sleep. These alternations were associated with an increase in breathing rate, respiratory variability, a depression of inspiratory related activity in genioglossus muscle and an increase in expiratory-related abdominal muscle activity when comparing deactivated (SWS-like) to activated (REM-like) states. These results demonstrate that urethane anesthesia consistently induces sleep-like brain state alternations and correlated changes in respiratory activity across different rodent species. They open up the powerful possibility of utilizing transgenic mouse technology for the advancement and translation of knowledge regarding sleep cycle alternations and their impact on respiration.

## Introduction

Sleep is a fundamental circadian behavior that has fascinated scientists for centuries. Although its functional role is still enigmatic, sleep has a powerful influence on behavior, mental health, cognition, and different physiological functions in vertebrates [1–4].

Sleep is not a unitary process. It involves systematic transitions between different levels of brain activity. The initiation of sleep is characterized by the gradual development of high-amplitude low-frequency activity in the forebrain and lowered muscle tone (non-REM or slow wave sleep: SWS) that eventually progresses into a paradoxical brain state characterized by low voltage fast frequency activity in the cortex and hippocampal theta activity accompanied by a generalized muscle atonia and rapid eye movements (REM).

The major anatomical brain structures that act as key players in the determination of the sleep/wake cycles have been identified (mesopontine nuclei, tuberomammillary bodies, locus coeruleus, raphe nucleus, hypothalamus) but the precise sequence of events that lead to brain state alternations and the mechanistic understanding of the process are still under debate [1,5]. The lack of an appropriate experimental model for sleep (other than sleep itself) is likely responsible for the difficult progress in this field.

Murine natural sleep is characterized by a very irregular and unpredictable sequence of events. Sleep cycles have been reported to be between 20 seconds and 13 minutes in length, with irregular occurrence of REM states during sleep that can last mere seconds to a few minutes [6–9]. Respiratory activity during sleep is characterized by a drop in ventilation and respiratory muscle activity during SWS followed by an increase in respiratory frequency and variability preferentially during REM sleep, although ventilatory control and respiratory characteristics can change considerably amongst different mouse strains [10–12].

The development of transgenic mouse lines provides further experimental power for sleep studies since different neuronal populations can be labelled, selectively activated, and permanently or reversibly silenced. This general approach may provide insight into the functional role and dynamics of structures that are involved in brain state alternations and in the modulation of respiratory networks, as well as provide new working hypotheses on disease physiology and enable mechanistic hypotheses to be tested at both cellular and network levels.

Recently, urethane anesthesia has been demonstrated to be a useful model of sleep dynamics in rats, where spontaneous brain state alternations at neocortical and hippocampal sites replicate, in a reproducible and predictable fashion, the spontaneous alternation between a SWS state and a REM state occurring in natural sleep [13]. In addition, it has also been demonstrated that under urethane, breathing shows changes correlated to these same brain state alternations that correspond to many of the typical respiratory features occurring during natural sleep and its stages [14].

Here we investigated the possibility that similar changes in forebrain activity and respiration occur in adult mice anesthetized with urethane. We show that, similar to rats, mice under urethane show spontaneous brain state alternations and correlated respiratory changes that also mimic sleep patterns. This confirms the potential of urethane anesthesia as a model for sleep across different rodent species and opens up a novel model system for determining mechanistic processes at a molecular and genetic level for sleep-like brain state changes and their physiological correlates.

## Materials and Methods

Animal handling and experimental protocols were approved by the Biosciences and Health Science Animal Policy and Welfare Committees of the University of Alberta according to the guidelines established by the Canadian Council on Animal Care.

### Acute (urethane-anesthetized) mice preparation

Thirteen adult male mice (7 CD1 mice and 6 C57BL/6 mice; 25-60g; mean=40.2±4.5g; no statistical difference in weight between the 2 strains, CD1= 47.4±6.3; C57BL/6 33.0±5.1; p=0.11) were used for acute experiments in this study. Mice were initially anesthetized with isoflurane (2% in 100% O_2_) while the femoral vein was cannulated and urethane (~1.5 g/kg body weight) was gradually delivered i.v. to induce anesthesia. In some cases, when the intravenous route became blocked or proved difficult, urethane was administered or supplemented intraperitoneally to a similar final dosage. Additional doses of anesthesia (at 0.2g/ml concentration in 0.01ml amounts) were delivered as necessary to maintain a surgical plane of anesthesia which was verified on occasion during experiments by lack of reaction to hindpad pressure or other noxious stimuli such as tail pinches. We ensured, as shown previously for rats [13], that these stimulations did not alter the rhythmicity or phase of subsequent brain state alternations. Additional saline (0.1ml) was delivered subcutaneously every two hours in order to maintain hydration levels. A servo-controlled heating pad was set at 36-37±1°C to maintain body temperature (model 50-7220, Harvard Apparatus) and rectal temperature was continuously recorded during a subset of experiments (n=4).

Bipolar EMG wire electrodes made with multi strand, Teflon-coated, stainless steel wires (Cooner Wire, Chatsworth, CA) were inserted into the genioglossus (GG), oblique abdominal (ABD), and intercostal muscles (INT) to measure breathing characteristics. For GG_EMG_ placement, a skin incision was performed under the chin to expose the digastric muscle which lies above the mylohyoid and genioglossus muscles. Insulated wires with a 1mm length exposure were then inserted through the genioglossus muscle at 1-2mm distance from each other. For ABD_EMG_ placement, a skin incision was performed on the right side of the abdomen, just below the rib cage and bipolar electrodes were placed in the oblique ABD muscle at 1-2mm distance from each other. Skin incisions were then sutured back to avoid drying of the muscles. For INT_EMG_ placement, a skin incision was performed on the right side of the chest and the pectoral muscle was displaced in order to expose the intercostal muscles. Bipolar electrodes were placed in the intercostal muscles at 1-2mm distance from each other. Skin was then sutured back together. Bipolar signals were amplified at 10,000X gain and filtered between 100 and 20kHz using a differential amplifier (model 1700, AM-Systems Inc, Carlsborg, WA).

Mice were then positioned on a stereotaxic frame and bipolar electrodes made with Teflon-coated stainless steel wires (~1mm vertical stagger; AM-Systems Inc.) were implanted in the hippocampal formation (HPC) according to the following coordinates relative to bregma: anterior-posterior (AP): -2.0; mediolateral (ML): ±1.5; dorsoventral (DV): -1.5 to -2.0mm. Local field potential (LFP) signals were amplified across contacts at 1000X gain and filtered between 0.1 and 10kHz bandwidth using the same differential amplifiers as for EMG. Hippocampal electrodes were lowered in the DV axis just below the position where a marked increase in multiunit discharge was detected via an audio amplifier (model 3300, AM-systems, Inc) and a prominent theta rhythm could be observed in the LFP. This corresponded to a relative position of both poles of the electrode across the pyramidal layer of CA1. Electrodes were then fixed to the skull by jeweler’s screws and dental acrylic.

Mice were allowed to stabilize for an hour before recordings took place. All recordings were made in a small, quiet, and artificially lit room and were always made while mice were positioned (using indwelling ear bars) in the stereotaxic apparatus which was oriented away from the centre of the room on a vibration proof table. They were spontaneously breathing room air. Thus, they had extremely limited (and virtually unchanging) external visual, auditory, somatosensory, and olfactory stimulation. All signals were sampled at 2 kHz following automatic antialiasing filtering using a PowerLab 16/30 data acquisition system (A D Instruments Inc., Colorado Springs, CO). At the end of the experiment, mice were euthanized by urethane overdose.

### Data analysis

During urethane anesthesia, REM-like and SWS-like states were identified by performing spectrographic analysis on moving 6s intervals and calculating the percentage of total power at specific frequencies in LabChart Pro7 software. In this set of experiments, we used hippocampal LFPs in order to clearly identify the activated (i.e., REM-like) state by the expression of activity in the theta frequency range (3-4Hz) and the deactivated state (e.g. SWS-like) by the expression of the slow oscillation (~1Hz). Since spontaneous brain state alternations can be observed simultaneously from both cortical and hippocampal recording sites in rats, measurements in either site provide an accurate identification of forebrain state [13–15]. Indeed, we have found in these earlier studies that hippocampal theta provides a more sensitive index of forebrain activation. Since hippocampal activated (REM-like) states are characterized by a 3-4Hz theta rhythm, this state was defined when the power in the HPC recordings at the 3-4 Hz interval was ≥ 40% of total power for 3 consecutive 6 s intervals (FFT size 16K, Cosine Bell data window with 25% overlap). Conversely, a deactivated (SWS-like) state was defined when hippocampal power in the 0.2-1.2 Hz bandwidth was ≥40% of total power for 3 consecutive 6 s intervals. Periods that did not meet these two conditions (when power in the 3-4 Hz and 0.2-1.2 Hz bandwidths was < 40% of total power) were identified as transition states. Transition states have also been characterized during both urethane anesthesia and natural sleep in rodents as demonstrating transient (less than 0.5s in duration) neocortical oscillatory (7–15 Hz) spindle events, consistent with activity during stage 2 nREM sleep [13].

The rhythmic interaction of forebrain state alternations and fluctuations in body temperature, previously shown to be linked in urethane-anesthetized rats [16], were assessed in mice with continuous recordings of temperature (n=4). For this purpose, brain state was characterized by the log ratio of power in the theta and slow oscillation ranges from hippocampal LFP recordings. Using custom Matlab code (V 5.3, The MathWorks, Natick, MA), both power and temperature signals were down sampled to 10Hz, detrended and normalized to have peak to peak amplitudes of 1. Both autocorrelation and cross correlation functions were assessed on these processed signals, again in Matlab. The average period was calculated from the time of the first positive peak at non zero lag in the autocorrelation function and the lag across signals was similarly calculated from the time of the first positive peak closest to the zero lag in the cross correlation function. Phase angles were calculated by dividing the time lag across signals by the period of the alternations and multiplying by 360.

Raw respiratory muscle EMG activity was rectified and integrated (time constant decay 0.08s); baseline and peak amplitude was calculated with the peak analysis application in LabChart7 Pro software. Respiratory period was obtained by measuring the time between two consecutive peak inspiratory events. In order to measure respiratory variability we obtained the coefficient of variation of the period by calculating the standard deviation of the respiratory period divided by its mean for each state across experiments.

Sighs were identified by the presence of an augmented respiratory effort (>50% increase in integrated GG_EMG_ peak amplitude in comparison to a regular breath) [17–20].

Single values were averaged according to the state and 1-tail paired t tests (for measurements across different states) and 2-tail unpaired t tests (for comparisons across the two mice strains) were used to determine significance of results. Data values are reported as mean ± standard error of the mean.

## Results

### Brain state alternations in mice under urethane anesthesia

**Figure 1:**
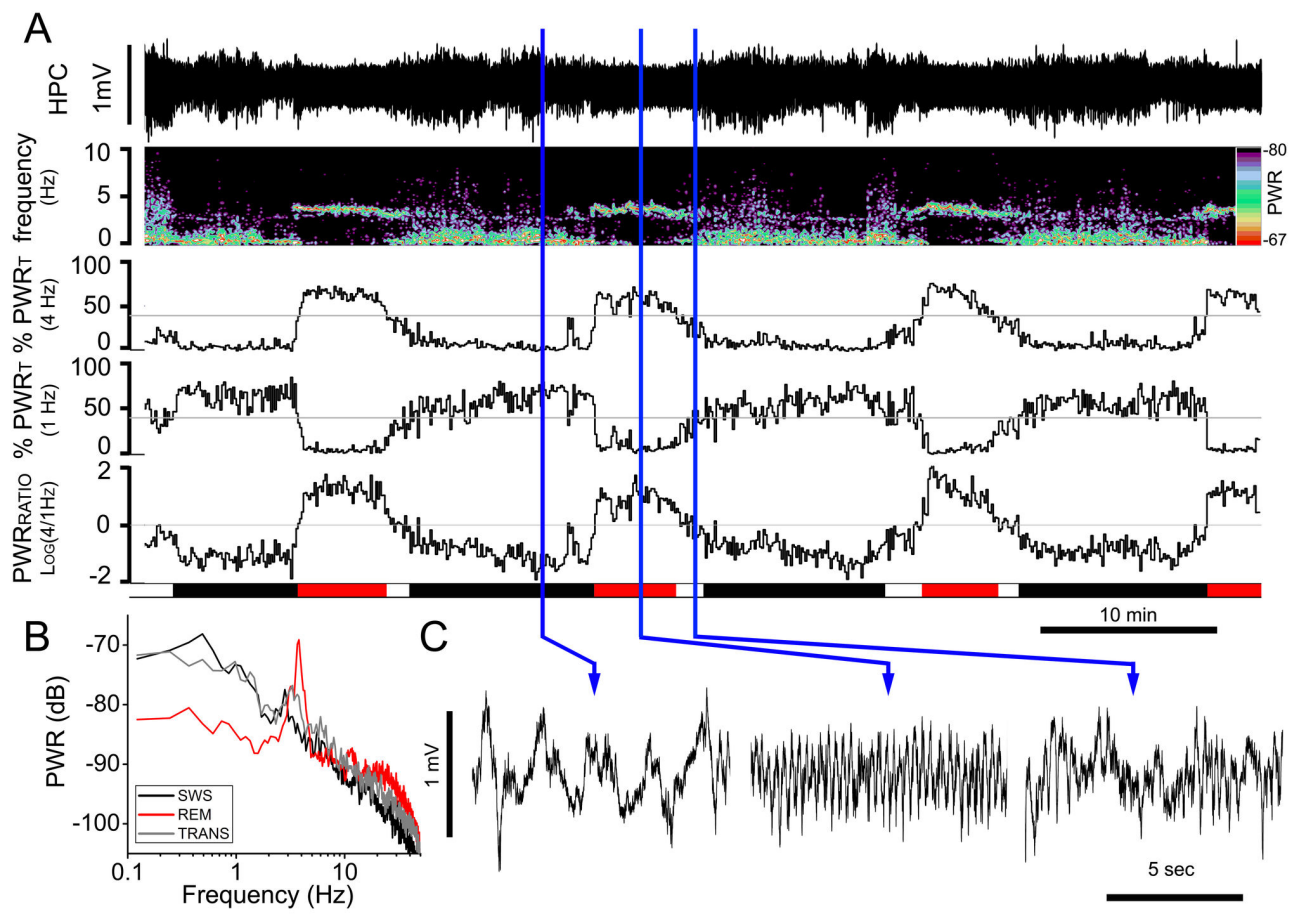
Spontaneous brain state alternations in urethane anesthetized mice. A) Long-term EEG recording of hippocampal activity (HPC) and corresponding power spectrogram (1-10Hz frequency range) during spontaneous brain state alternations under urethane anesthesia. Below, the percentage of relative power (% PWR_T_) in the 3.5-4.5 and 0.2-1.2 Hz intervals and their ratio (on log scale) are displayed. Grey lines at the 40% of relative PWR_T_ indicates the cutoff values for the identification of different states (REM-like, transition, SWS-like). Schematic blocks at the bottom of the plot indicate the time spent in REM-like (red), transition (white) and SWS-like (black) states. B) Power spectral analysis for HPC activity during SWS-like, REM-like and transition states further indicate the prevalence of ~1Hz power during SWS-like state and the prevalence of ~4Hz power during REM-like events. Note the increase in gamma band (25-40 Hz) frequency during REM-like state compared to SWS-like state. C) Magnification of the raw HPC traces in SWS-like (left), REM-like (centre) and transition (right). Blue vertical lines indicate the time point from which the traces are taken.

Similar to what has been previously demonstrated in adult rats [13–15], 10 of 13 mice under urethane anesthesia displayed clear spontaneous brain-state alternations (Figure 1). In the 3 remaining mice, two were excluded for poor quality LFP signals that made automated determination of brain state difficult (although brain state alternations were apparent visually) and one did not display any systematic alternations despite good quality recordings. In total, we analyzed 24.33 hours of spontaneous recordings from 10 mice (5 CD1 and 5 C57BL/6). There were no significant differences in the various parameters extracted from any EEG and respiratory signal between the two strains (p>0.05 in all t-test between strains), therefore we report data both for individual strains and pooled data (Table 1).

**Table 1:** EEG and respiratory characteristics in CD1 and C57BL/6 mice across states.

	CD1	C57BL/6	CD1 +C57BL/6
Cycle length (min)	11.22±2.11 (5)	16.15±1.32 (5)	13.90±1.38 (10)
REM-like state (min)	3.35±0.63 (28.9±5.1%)	4.37±0.73 (27.7±6.1%)	3.85±0.48 (28.3±3.7%)
Transition state (min)	2.50±0.45 (21.6±3.7%)	3.08±0.47 (18.1±1.6%)	2.78±0.32 (19.8±1.9%)
SWS-like state (min)	5.98±1.48 (49.5±7.6%)	9.25±1.47 (54.2±4.8%)	7.62±1.12 (51.9±4.3%)
REM-like resp. period (s)	0.40±0.03 (5)	0.42±0.03 (5)	0.41±0.02 (10)
Transition resp. period (s)	0.41±0.02	0.47±0.04	0.44±0.02
SWS-like resp. period (s)	0.42±0.02	0.48±0.04	0.45±0.02
REM-like CV period (s)	0.20±0.06 (5)	0.28±0.05 (5)	0.24±0.04 (10)
Transition CV period (s)	0.16±0.04	0.23±0.06	0.19±0.03
SWS-like CV period (s)	0.19±0.05	0.17±0.04	0.18±0.03
REM-like sigh/hr	45.9±7.5 (5)	57.5±9.0 (5)	51.7±5.8 (10)
Transition sigh/hr	40.1±8.4	46.9±11.6	43.5±6.8
SWS-like sigh/hr	39.9±9.2	27.9±6.4	33.9±5.6
∫INTpeak (REM/SWS)	1.01±0.04 (5)	0.95±0.09 (5)	0.98±0.04 (10)
∫INTpeak (REM/trans)	1.01±0.02	0.91±0.03	0.97±0.03
∫GGpeak (REM/SWS)	0.88±0.03 (3)	0.79±0.05 (5)	0.82±0.04 (8)
∫GGpeak (REM/trans)	0.94±0.04	0.85±0.03	0.88±0.03
∫ABDpeak (REM/SWS)	1.19±0.14 (2)	1.99±0.24 (2)	1.60±0.26 (4)
∫ABDpeak (REM/trans)	1.09±0.05	1.34±0.08	1.22±0.08

Average data for brain state alternations and respiratory characteristics according to mice line (CD1, C57BL/6 or pooled data). Percentages in brackets indicates the percentage of time spent in each cycle. Number in italics indicates the number of mice used.

Brain activity displayed quasi-rhythmic transitions between an activated (REM-like) state and a deactivated (SWS-like) state. The REM-like state was characterized by high power in the theta frequency bandwidth (range: 3.5-4.5 Hz) with a corresponding increase in power through the gamma band (25-40Hz), consistent with the expression of gamma activity during both natural sleep and during anesthesia in previous studies [21,22]. The SWS-like state was characterized by high power slow oscillation (SO) bandwidth (range: 0.2-1.2 Hz). In between these two states, a transition period was identified which exhibited intermediate electrographic characteristics (<40% power in both theta and SO frequency bands).

The average duration (period) of state alternations was 13.90±1.38 min (CD1= 11.22±2.11min, n=5; C57BL/6= 16.15±1.32min, n=5; p=0.08; table 1), again comparable to that shown in rats [13]. During these alternations, the average time spent in the activated (REM-like) phase was 3.85±0.48 min which constituted 28.3±3.7% of cycle time (CD1=3.35±0.63min; C57BL/6=4.37±0.73min; p=0.32). For the transition phase the average duration was 2.78±0.32min which constituted 19.8±1.9% of cycle time (CD1=2.50±0.45min; C57BL/6=3.08±0.47min; p=0.40) and finally 7.62±1.12 min or 51.9±4.3% of cycle time was spent in the deactivated (SWS-like) phase (CD1=5.98±1.48min; C57BL/6=9.25±1.47 min; p=0.16).

Once mice developed spontaneous brain state alternations, the state changes persisted for several hours, further supporting the hypothesis that the alternations are intrinsic to the anesthetic effect induced by urethane and not generated by a gradual waning of the anesthetic level. Indeed, as previously confirmed in rats [13], we ensured that mice were always at a surgical plane of anesthesia, regardless of state. Although variable in timing across animals, the fluctuations in power and the period at which they alternated tended to be consistent within the same animal with occasional transient shortenings. Since our conditions were always similar across animals, it is unlikely that this variability was due to environmental conditions.

**Figure 2:**
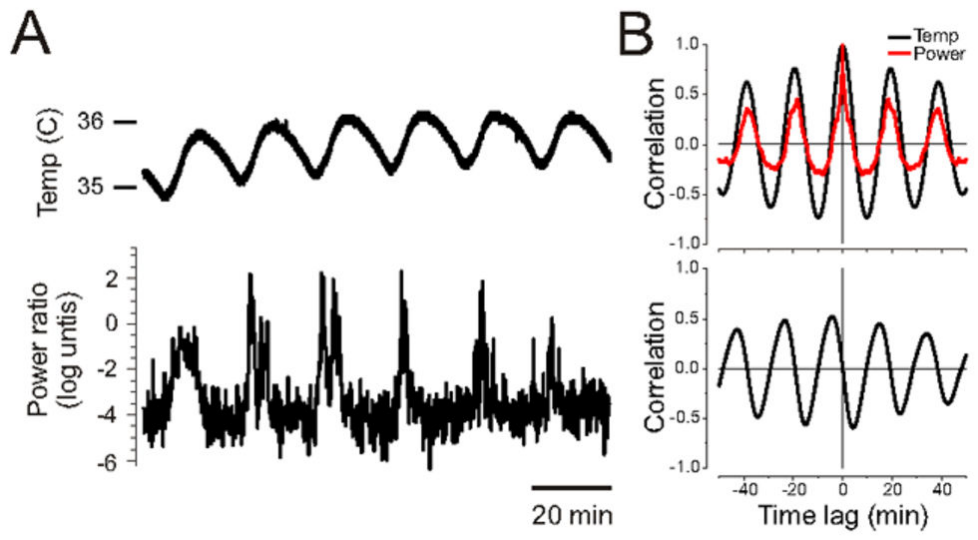
Spontaneous brain state alternations are coordinated with fluctuations in body temperature. A) Core temperature measurements (upper trace) and state alternations, as shown by fluctuations in theta/slow oscillation (4Hz/1Hz) power ratios in hippocampal LFP (lower trace), appear rhythmically coordinated over a long recording period. B) Autocorrelation function (top panel) of traces shown in A) demonstrate rhythmicity of both temperature (black) and power (red) fluctuations occurring at a similar period of approximately twenty minutes. Cross correlation function (lower panel) between temperature and power measures shows coupling between these rhythms with peak in temperature lagging behind that of power by slightly over 4 minutes.

In a subgroup of mice (n=4), we simultaneously measured core body temperature in order to evaluate any coupling between this measure and brain state alternations (Figure 2). As previously shown in rats [16], rhythmic coupling was observed in all cases (albeit in 1 case, the coupling was weaker and transient across samples). This was observed from the similar period of fluctuations of power and temperature as shown by autocorrelation functions and by a parallel rhythmicity in the cross correlation function. Cross correlation functions between power ratios describing forebrain state alternations and body temperature showed that temperature peaks lagged behind those of power at an average of 3.68 ± 0.62 minutes (corresponding to an average Raleigh phase angle of 69.7^°^). Interestingly, this coupling was strongest for experiments in which the period of state alternations was extended. Indeed, in the above data, the average period of state alternations was 19.15±0.37 minutes.

**Figure 3:**
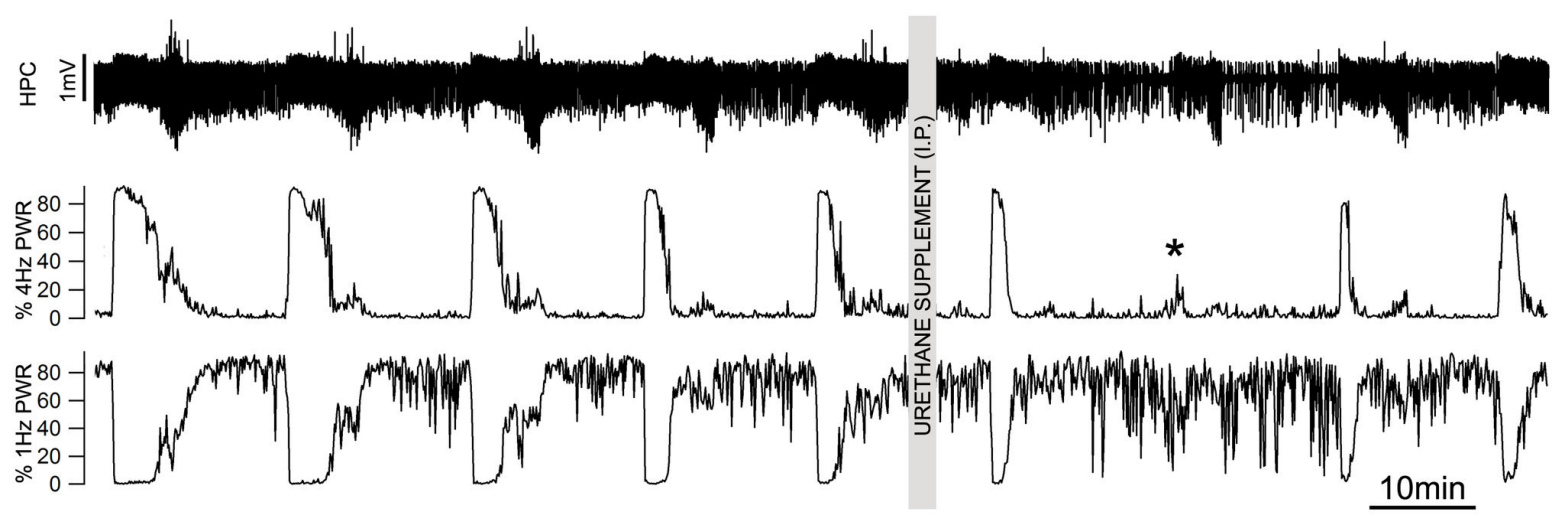
Supplemental doses of urethane anesthesia do not affect rhythmicity of brain state alternations. Long term EEG recording at the HPC site and its corresponding percentage of total power calculated in the 3-4Hz range and for 0.2-1.2Hz range during regular brain state transitions under urethane anesthesia and supplemental dose of urethane (0.02ml at 0.2 g/ml dose). Note the temporary depression of power in the theta range (asterisk) followed by the resumption of regular brain state alternations.

In a further subgroup of six mice (4 C57BL/6 and 2 CD1mice), we also assessed the influence of additional doses of anesthesia on cyclic state alternations (Figure 3). In four animals, supplemental doses of anesthesia (2-4mg -0.01-2ml) did not affect the duration nor the periodicity of brain state alternations (15.6±5.4 versus 16.0±5.1 minutes, p=0.38) but did significantly reduce the time spent in REM-like state (from 28.1±2.5% to 15.4±2.6%, p=0.04). Although increases were observed in both SWS-like (55.8±6.0% to 64.3±5.2%; p=0.18) and transition states (17.4±6.2% to 22.7±4.5%; p=0.4) neither of these latter changes achieved statistical significance (Figure 3). In addition to a significant decrease in the duration of the activated state, the proportion of 4Hz power during REM-like states decreased to 56.1±10.2% (p=0.048) of pre infusion values. In a further two experiments, additional doses of anesthesia temporarily disrupted brain state alternations (with maintenance of a persistent SWS-like state for 32.8 and 50.13 min, respectively. After this time, state transitions then resumed at a similar period (14.2±4.2 versus 15.7±1.6 min, p=0.5).

### Breathing shows state dependencies in mice under urethane anesthesia

**Figure 4:**
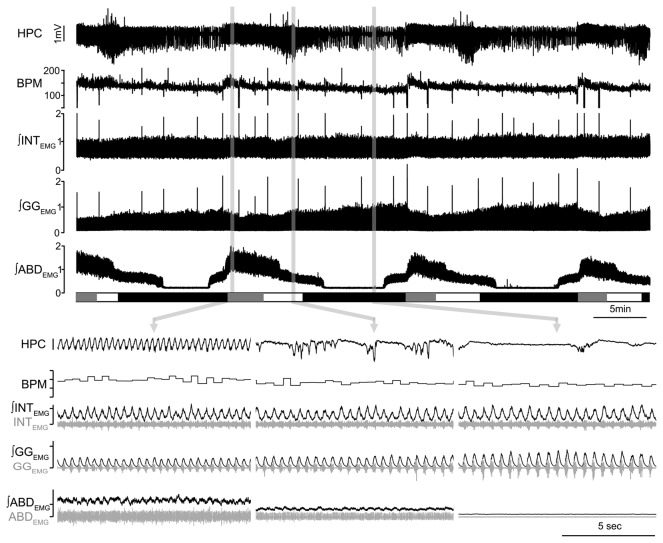
State dependent modulation of breathing in urethane anesthetized mice. A) Long-term EEG recording at the HPC site and corresponding respiratory frequency (breath per minute, BPM) and rectified and integrated activity of intercostals (INT), genioglossus (GG) and abdominal (ABD) muscle EMG activity across spontaneous brain state alternations. Schematic block at the bottom of the plot indicate time spent in REM-like (grey), transition (white) and SWS-like (black) states. B) Details of the HPC trace, BPM and raw and integrated traces of INT, GG and ABD EMG in REM-like (left), transition (centre) and SWS-like states.

A variety of respiratory measures are strongly correlated to brain state changes under urethane anesthesia in adult rats [14] in a fashion that reproduces breathing characteristics during natural sleep in both humans and rats [14,23–27]. We therefore investigated if respiratory parameters are also affected by brain state alternations in urethane anesthetized mice (Figure 4 table 1).

The average respiratory period across states was 0.41±0.02s (CD1=0.40±0.03s, n=5; C57BL/6=0.42±0.03s, n=5; p=0.60), 0.44±0.02s (CD1=0.41±0.02s; C57BL/6=0.47±0.04s; p=0.18) and 0.45±0.02s (CD1=0.42±0.02s; C57BL/6=0.48±0.04s; p=0.24) for REM-like, transition and SWS-like states, respectively (corresponding to frequencies of 149.03±7.14, 139.4±6.7 and 137.0±6.9 breaths per minute, respectively; n=10). In a within-subjects comparison, the respiratory period was significantly shorter (i.e. faster) in the REM-like state in comparison to both transition (p=8.8x10^-3^) and SWS-like (p=4.9x10^-3^) states. Respiratory variability, as measured by the coefficient of variation of the respiratory period, was higher in the REM-like state (0.24±0.04; CD1= 0.20±0.06; C57BL/6=0.28±0.05; strain comparison p=0.33) in comparison to both transition (0.19±0.03; p=0.025; CD1=0.16±0.04; C57BL/6=0.23±0.06; strain comparison p=0.34) and SWS-like states (0.18±0.03, p=0.016; CD1=0.19±0.05; C57BL/6=0.17±0.04; strain comparison p=0.83; n=10). The occurrence of sighs, augmented breaths characterized by an increased (>50%) inspiratory effort in both diaphragm, INT, and GG muscles that significantly increases tidal volume [20], was also higher during the REM-like state (51.7±5.8 sighs/hr; CD1=45.9±7.5; C57BL/6=57.5±9.0; strain comparison p=0.35) in comparison to both transition (43.5± 6.8sighs/hr p=0.03; CD1= 40.1±8.4; C57BL/6=46.9±11.6; strain comparison p=0.64) and SWS-like (33.9±5.6 sighs/hr; p=0.01; CD1= 39.9±9.2; C57BL/6=27.9±6.4; strain comparison p=0.31) states. Reduced period, increased variability and sigh rate during REM-like states are also typical breathing characteristics of urethane anesthetized rats [14] and of natural REM sleep in both humans and rodents [23–27].

The activity of INT (n=10), GG (n=10) and ABD (n=7) muscles was also monitored by using bipolar EMG electrodes (Figure 4). Integrated peak amplitude of INT_EMG_ was not affected by states (-3.1±3.1% decrease REM-like versus transition, p=0.08; -1.8±4.8% decrease REM-like versus SWS-like, p=0.22; see table for details of changes across states in the 2 strains, p>0.05).

When active, GG_EMG_ was consistently rhythmic and in phase with inspiration (Figure 4). In 8 out of 10 mice, the integrated amplitude of GG_EMG_ activity was significantly modulated by state. Integrated peak amplitude of GG_EMG_ during REM-like states was reduced on average to 82.3±4.0% (p=6.6x10^-3^) of SWS-like state values and to 88.5±3.2% (p=0.04) of transition state values (n=8). In one of the remaining mice the amplitude of GG_EMG_ activity remained consistent across brain states and was rhythmically related to inspiration whereas in another mouse we could not detect any respiratory related signal. These changes are consistent with those observed in rats during both natural sleep and under urethane anesthesia [14,28,29].

ABD_EMG_ activity was either absent (n=3) or had expiratory-related rhythmicity (n=4). In these latter mice, ABD activity displayed state dependent modulation, with integrated peak activity in REM-like state that was increased by 22.1±8.2% (p=0.02) as compared to transition states and increased by 59.6±25.6% (p=0.03) as compared to SWS-like states (Figure 3). This was again consistent with previous work performed in adult rats, where, although variable in occurrence, expiratory ABD_EMG_ activity displayed a prominent association with state in both urethane anesthesia and natural sleep [14].

In contrast to the above changes described for spontaneous state alternations, we additionally found that there was a marked difference in EMG activity elicited by a noxious stimulus delivered to the pad or tail which concomitantly induced a transient forebrain activation of hippocampal EEG. In both GG and INT traces, the delivery of this activating stimulus was associated with a modest increase in muscle tone. This suggests that brain activation achieved by noxious stimuli is not identical to that elicited spontaneously.

## Discussion

### Urethane anesthesia

Urethane anesthesia has been widely used for a variety of physiological, and especially neurophysiological, experimental paradigms. Despite a relatively common usage, its exact mechanism of anesthetic action is still unknown. Urethane is considered to be a non-typical general anesthetic due to its only moderate effects on both excitatory and inhibitory neurotransmission [30] in contrast to other commonly used veterinary/scientific anesthetics (isoflurane, propofol, ketamine, and barbiturates), which typically display a primary action of promoting inhibitory GABAergic neurotransmission, reducing excitatory glutamaterigic transmission, or both [31–34]. One of urethane’s most prominent cellular effects is the hyperpolarization of cortical neurons via potentiation of a resting potassium conductance [35]. Interestingly, modulation of potassium conductances that are active in the sub-threshold range (albeit different varieties) has been proposed to be important for producing a “sleepless” Drosophila mutant [36] and in the neurophysiological actions of adenosine, a classic and naturally occurring somnogenic agent [37]. This leads to the speculation that modulation of intrinsic membrane excitability as opposed to actions on either excitatory or inhibitory synaptic transmission may be a common mechanism for the maintenance of unconsciousness during both urethane anesthesia and natural sleep.

Although our group was not the first to describe spontaneous brain state alternations under urethane anesthesia, a common and continuing mis-interpretation is that the activated state is indicative of a reduced (wake-like) level of anesthesia [38–41]. We previously, and conclusively, demonstrated that this is not the case with urethane-anesthetized rats [13]. Across brain state alternations, there was no change in the level of responsiveness to a painful stimulus nor was there an increase in tonic skeletal muscle tone when transitioning to activated states. Indeed spontaneous activated states under urethane, as with natural REM, were often characterized by a reduction in tonic skeletal muscle tone [13]. This is in contrast to the effects of delivering noxious stimuli to urethane anesthetized animals that can also activate the forebrain but that *increase* skeletal (as well as GG and INT_EMG_ respiratory) muscle tone. In the present study, severe tail or hind paw pressure could activate both forebrain state and GG/INT_EMG_, but spontaneous changes into activated states showed the opposite profile on GG_EMG_ activity and had no effects on INT_EMG_. Thus, given the host of other physiological variables that also parallel changes across the sleep cycle including temperature and ventilation [14,16], as well as the influence of pharmacological and brainstem manipulations known to influence sleep states [13] we conclude that the spontaneous alternations present under urethane most closely resemble the natural sleep cycle between REM and SWS.

In a previous study [16] we discovered that thermal manipulations, similar to natural sleep, had a significant impact on brain state in urethane-anesthetized rats and could be an entraining influence upon their alternations. We also showed that despite this influence, brain state alternations and temperature fluctuations could be shown to be mutually independent, although they are likely coupled biological oscillators. In the present study, we took great care to ensure that any external fluctuations of heating were minimized by using a homeostatic system without marked feedback cycling. Even with the use of this system, like with our previous results in rats, we were able to demonstrate a significant and strong sleep-like coupling of brain state alternations to core temperature fluctuations in a majority of experiments.

Our present results show that spontaneous transitions are not simply a species-specific reaction of rats to urethane anesthesia but that they also occur in mice. Similar to rats, the timing of brain state alternations is comparable to spontaneous uninterrupted SWS/REM cycles occurring in natural sleep of mice [7,9]. Furthermore, although our sampling was limited (n= 5 in each case), we did not observe any significant differences in neurophysiological or respiratory measures between the two different mouse strains we used; the outbred CD1 and inbred C57BL/6 strain. These results contrast with the reported changes in ventilatory behavior across different inbred mice strains [10,42–44], although it has been shown that possible strain differences may be hampered by the use of urethane anesthesia [42]. We are not aware of any direct comparison of ventilator parameters between C57BL/6 and CD1 mice strains, however differences in neuroanatomy, behavior and pharmacological properties have been reported [45–48]. The current results suggest that mice under urethane anesthesia present a stereotyped alternation of brain state and respiratory behavior that makes differences between CD1 and C57BL/6 strains indistinguishable.

State dependent modulation of breathing in urethane anesthetized mice.

Similar to what we reported in adult rats [14], we observed obvious state dependent changes in several respiratory parameters in mice. In contrast to our previous results in rats, transition states (characterized by weak power in the hippocampus at both 4Hz and 1Hz frequencies) displayed respiratory characteristics more similar to the SWS-like states than REM-like states. This phenomenon may be due to differences in the intrinsic properties of the neuronal networks that control respiratory parameters in mice. Nonetheless, in mice, respiratory rate and respiratory variability were significantly increased during REM-like states compared to SWS-like and transition states. These changes reproduced what happens in natural sleep of both humans [23–27] and rodents [10,12–14,49]. Sigh rate was higher in REM-like state versus SWS-like state, similar to what we observed in urethane anesthetized rats and what is observed in natural sleep in humans [25–27] and in rats [14]. Interestingly, very few studies have analyzed sigh frequency in mice across sleep states and they reported a reduction in sigh/hr during REM state compared to SWS [12,50]. This discrepancy between human and rat natural sleep data and our urethane anesthetized data in mice may be due to the very limited time mice spend in REM epoch (≤ 2min) [7,8] which makes sigh rate calculations extremely challenging or impossible in addition to the fact that sighs can often occur at the transitions between SWS and REM states [14].

When the activity of different respiratory muscles was examined, including INT, GG, and ABD, we detected systematic differences across states as well. For INT muscle activity there was no significant change across states, similar to our previous results in urethane anesthetized rats [14] and naturally sleeping rats [49,51], where both diaphragm and INT muscles do not show significant and consistent changes in EMG activity across states. However, reduced activity in external INT muscle is sometimes observed in humans during REM compared to SWS states [52].

The activity of the GG muscle was consistently reduced in REM-like epochs compared to SWS-like and transitions states, similar to what happens in natural sleep of humans and rodents, where both tonic and respiratory modulated activity of the GG muscle progressively declines as sleep progresses from wakefulness to SWS and REM sleep [14,28,29,53–55]. We are not aware of studies on GG_EMG_ activity in unanesthetized mice across sleep/wake cycles, but, as demonstrated by activity of the correlated masseter muscle in mice and humans [56,57], we are quite confident that our data on GG_EMG_ activity in urethane anesthetized mice replicates the state dependent modulation that is present in humans, rats, and very likely in mice. These results further support the opportunity of using urethane anesthesia for the study of mechanisms governing state dependent modulation of GG activity in mice as well.

Similar to our results in both natural sleep and under urethane anesthesia [14], we observed occasional increases in expiratory-related muscle activity at the level of ABD muscles. These results suggest that in the activated states of urethane anesthetized mice, expiratory related activity may also be recruited in order to favor ventilation when inspiratory activity is more irregular and variable.

These results support the hypothesis that under urethane anesthesia, state dependent modulation of respiratory muscles occurs in a fashion that reproduces what happens in natural sleep, therefore allowing for mechanistic analysis of respiratory muscle control in an anesthetized preparations that display spontaneous and predictable changes that replicate phenomena occurring during natural sleep.

In conclusion, the observation that urethane anesthesia recapitulates natural sleep-like alternations in mice as well as in rats, opens the possibility of applying this model of sleep to study cellular and network mechanisms in different transgenic mice that display aberrant brain activity, sleep pattern and/or respiratory disturbances, such as mouse models for respiratory disorders (congenital central hypoventilation syndrome, Prader Willi syndrome) or transgenic mice that display either constitutive or inducible activation or silencing of specific neurons involved in brain state alternations or in respiratory control across states.

## References

[1] Brown RE, Basheer R, McKenna JT, Strecker RE, McCarley RW (2012) Control of sleep and wakefulness. Physiol Rev 92: 1087-1187. doi:10.1152/physrev.00032.2011. PubMed: 22811426. 22811426 10.1152/physrev.00032.2011 PMC3621793

[2] Havekes R, Vecsey CG, Abel T (2012) The impact of sleep deprivation on neuronal and glial signaling pathways important for memory and synaptic plasticity. Cell Signal 24: 1251-1260. doi:10.1016/j.cellsig.2012.02.010. PubMed: 22570866. 22570866 10.1016/j.cellsig.2012.02.010 PMC3622220

[3] Trinder J, Jordan AS (2012) Activation of the upper airway dilator muscle genioglossus during sleep is largely dependent on an interaction between chemical drive and mechanoreceptor feedback. Sleep 34: 983-984. PubMed: 21804659. 10.5665/SLEEP.1144 PMC3138171 21804659

[4] Barone MT, Menna-Barreto L (2011) Diabetes and sleep: a complex cause-and-effect relationship. Diabetes Res Clin Pract 91: 129-137. doi:10.1016/j.diabres.2010.07.011. PubMed: 20810183. 20810183 10.1016/j.diabres.2010.07.011

[5] Saper CB, Fuller PM, Pedersen NP, Lu J, Scammell TE (2010) Sleep state switching. Neuron 68: 1023-1042. doi:10.1016/j.neuron.2010.11.032. PubMed: 21172606. 21172606 10.1016/j.neuron.2010.11.032 PMC3026325

[6] Douglas CL, Bowman GN, Baghdoyan HA, Lydic R (2005) C57BL/6J and B6.V-LEPOB mice differ in the cholinergic modulation of sleep and breathing. J Appl Physiol 98: 918-929. PubMed: 15475596. 15475596 10.1152/japplphysiol.00900.2004

[7] Tsubone H, Sawazaki H (1979) Electroencephalographic patterns and their alternating process in mice. Nihon Juigaku Zasshi 41: 495-504. 513396 10.1292/jvms1939.41.495

[8] Weiss T, Rokdán E (1964) Comparative study of sleep cycles in rodents. Experientia 20: 280-281. doi:10.1007/BF02151809. PubMed: 5856319. 5856319 10.1007/BF02151809

[9] Van Twyver H (1969) Sleep patterns of five rodent species. Physiol Behav 4: 901-905. doi:10.1016/0031-9384(69)90038-9.

[10] Friedman L, Haines A, Klann K, Gallaugher L, Salibra L et al. (2004) Ventilatory behavior during sleep among A/J and C57BL/6J mouse strains. J Appl Physiol 97: 1787-1795. doi:10.1152/japplphysiol.01394.2003. PubMed: 15475556. 15475556 10.1152/japplphysiol.01394.2003

[11] Han F, Subramanian S, Price ER, Nadeau J, Strohl KP (2002) Periodic breathing in the mouse. J Appl Physiol 92: 1133-1140. PubMed: 11842050. 11842050 10.1152/japplphysiol.00785.2001

[12] Nakamura A, Fukuda Y, Kuwaki T (2003) Sleep apnea and effect of chemostimulation on breathing instability in mice. J Appl Physiol 94: 525-532. doi:10.1063/1.1582236. PubMed: 12433867. 12433867 10.1152/japplphysiol.00226.2002

[13] Clement EA, Richard A, Thwaites M, Ailon J, Peters S et al. (2008) Cyclic and sleep-like spontaneous alternations of brain state under urethane anaesthesia. PLOS ONE 3: e2004. doi:10.1371/journal.pone.0002004. PubMed: 18414674. 18414674 10.1371/journal.pone.0002004 PMC2289875

[14] Pagliardini S, Greer JJ, Funk GD, Dickson CT (2012) State-dependent modulation of breathing in urethane-anesthetized rats. J Neurosci 32: 11259-11270. doi:10.1523/JNEUROSCI.0948-12.2012. PubMed: 22895710. 22895710 10.1523/JNEUROSCI.0948-12.2012 PMC6621193

[15] Wolansky T, Clement EA, Peters SR, Palczak MA, Dickson CT (2006) Hippocampal slow oscillation: a novel EEG state and its coordination with ongoing neocortical activity. J Neurosci 26: 6213-6229. doi:10.1523/JNEUROSCI.5594-05.2006. PubMed: 16763029. 16763029 10.1523/JNEUROSCI.5594-05.2006 PMC6675178

[16] Whitten TA, Martz LJ, Guico A, Gervais N, Dickson CT (2009) Heat synch: inter- and independence of body-temperature fluctuations and brain-state alternations in urethane-anesthetized rats. J Neurophysiol 102: 1647-1656. doi:10.1152/jn.00374.2009. PubMed: 19587317. 19587317 10.1152/jn.00374.2009

[17] Orem J, Trotter RH (1993) Medullary respiratory neuronal activity during augmented breaths in intact unanesthetized cats. J Appl Physiol 74: 761-769. PubMed: 8458793. 8458793 10.1152/jappl.1993.74.2.761

[18] Marshall JM, Metcalfe JD (1988) Cardiovascular changes associated with augmented breaths in normoxia and hypoxia in the rat. J Physiol 400: 15-27. PubMed: 3418526. 3418526 10.1113/jphysiol.1988.sp017107 PMC1191794

[19] Hoch B, Bernhard M, Hinsch A (1998) Different patterns of sighs in neonates and young infants. Biol Neonate 74: 16-21. doi:10.1159/000014006. PubMed: 9657665. 9657665 10.1159/000014006

[20] Bartlett D Jr. (1971) Origin and regulation of spontaneous deep breaths. Respir Physiol 12: 230-238. doi:10.1016/0034-5687(71)90055-7. PubMed: 5568463. 5568463 10.1016/0034-5687(71)90055-7

[21] Buzsáki G, Buhl DL, Harris KD, Csicsvari J, Czéh B et al. (2003) Hippocampal network patterns of activity in the mouse. Neuroscience 116: 201-211. doi:10.1016/S0306-4522(02)00669-3. PubMed: 12535953. 12535953 10.1016/s0306-4522(02)00669-3

[22] Kiss T, Feng J, Hoffmann WE, Shaffer CL, Hajós M (2013) Rhythmic theta and delta activity of cortical and hippocampal neuronal networks in genetically or pharmacologically induced N-methyl-D-aspartate receptor hypofunction under urethane anesthesia. Neuroscience 237: 255-267. doi:10.1016/j.neuroscience.2013.01.058. PubMed: 23396086. 23396086 10.1016/j.neuroscience.2013.01.058

[23] Aserinsky E, Kleitman N (1953) Regularly occurring periods of eye motility, and concomitant phenomena, during sleep. Science 118: 273-274. doi:10.1126/science.118.3062.273. PubMed: 13089671. 13089671 10.1126/science.118.3062.273

[24] Aserinsky E (1965) Periodic respiratory pattern occurring in conjunction with eye movements during sleep. Science 150: 763-766. doi:10.1126/science.150.3697.763-a. PubMed: 5844080. 10.1126/science.150.3697.763-a 5844080

[25] McNamara F, Lijowska AS, Thach BT (2002) Spontaneous arousal activity in infants during NREM and REM sleep. J Physiol 538: 263-269. doi:10.1113/jphysiol.2001.012507. PubMed: 11773333. 11773333 10.1113/jphysiol.2001.012507 PMC2290006

[26] Fukumizu M, Kohyama J (2004) Central respiratory pauses, sighs, and gross body movements during sleep in children. Physiol Behav 82: 721-726. doi:10.1016/j.physbeh.2004.06.011. PubMed: 15327922. 15327922 10.1016/j.physbeh.2004.06.011

[27] Qureshi M, Khalil M, Kwiatkowski K, Alvaro RE (2009) Morphology of sighs and their role in the control of breathing in preterm infants, term infants and adults. Neonatology 96: 43-49. doi:10.1159/000201738. PubMed: 19204409. 19204409 10.1159/000201738

[28] Megirian D, Hinrichsen CF, Sherrey JH (1985) Respiratory roles of genioglossus, sternothyroid, and sternohyoid muscles during sleep. Exp Neurol 90: 118-128. doi:10.1016/0014-4886(85)90045-7. PubMed: 4043287. 4043287 10.1016/0014-4886(85)90045-7

[29] Horner RL (2008) Neuromodulation of hypoglossal motoneurons during sleep. Respir Physiol Neurobiol 164: 179-196. doi:10.1016/j.resp.2008.06.012. PubMed: 18620080. 18620080 10.1016/j.resp.2008.06.012

[30] Hara K, Harris RA (2002) The anesthetic mechanism of urethane: the effects on neurotransmitter-gated ion channels. Anesth Analg 94: 313-318, table of contents. doi:10.1213/00000539-200202000-00015. PubMed : 11812690 11812690 10.1097/00000539-200202000-00015

[31] Antkowiak B (2001) How do general anaesthetics work? Naturwissenschaften 88: 201-213. doi:10.1007/s001140100230. PubMed: 11482433. 11482433 10.1007/s001140100230

[32] Franks NP (2006) Molecular targets underlying general anaesthesia. Br J Pharmacol 147 Suppl 1: S72-S81. PubMed: 16402123. 16402123 10.1038/sj.bjp.0706441 PMC1760740

[33] Thompson SA, Wafford K (2001) Mechanism of action of general anaesthetics--new information from molecular pharmacology. Curr Opin Pharmacol 1: 78-83. doi:10.1016/S1471-4892(01)00013-3. PubMed: 11712540. 11712540 10.1016/s1471-4892(01)00013-3

[34] Rudolph U, Antkowiak B (2004) Molecular and neuronal substrates for general anaesthetics. Nat Rev Neurosci 5: 709-720. doi:10.1038/nrn1496. PubMed: 15322529. 15322529 10.1038/nrn1496

[35] Sceniak MP, Maciver MB (2006) Cellular actions of urethane on rat visual cortical neurons in vitro. J Neurophysiol 95: 3865-3874. doi:10.1152/jn.01196.2005. PubMed: 16510775. 16510775 10.1152/jn.01196.2005

[36] Koh K, Joiner WJ, Wu MN, Yue Z, Smith CJ et al. (2008) Identification of SLEEPLESS, a sleep-promoting factor. Science 321: 372-376. doi:10.1126/science.1155942. PubMed: 18635795. 18635795 10.1126/science.1155942 PMC2771549

[37] Dunwiddie TV, Masino SA (2001) The role and regulation of adenosine in the central nervous system. Annu Rev Neurosci 24: 31-55. doi:10.1146/annurev.neuro.24.1.31. PubMed: 11283304. 11283304 10.1146/annurev.neuro.24.1.31

[38] Grahn DA, Heller HC (1989) Activity of most rostral ventromedial medulla neurons reflect EEG/EMG pattern changes. Am J Physiol 257: R1496-R1505. PubMed: 2690651. 2690651 10.1152/ajpregu.1989.257.6.R1496

[39] Hunter JD, McLeod JZ, Milsom WK (1998) Cortical activation states in sleep and anesthesia. II: respiratory reflexes. Respir Physiol 112: 83-94. doi:10.1016/S0034-5687(98)00020-6. PubMed: 9696285. 9696285 10.1016/s0034-5687(98)00020-6

[40] Hunter JD, Milsom WK (1998) Cortical activation states in sleep and anesthesia. I: Cardio-respiratory effects. Respir Physiol 112: 71-81. doi:10.1016/S0034-5687(98)00018-8. PubMed: 9696284. 9696284 10.1016/s0034-5687(98)00018-8

[41] Murakami M, Kashiwadani H, Kirino Y, Mori K (2005) State-dependent sensory gating in olfactory cortex. Neuron 46: 285-296. doi:10.1016/j.neuron.2005.02.025. PubMed: 15848806. 15848806 10.1016/j.neuron.2005.02.025

[42] Gonsenhauser I, Wilson CG, Han F, Strohl KP, Dick TE (2004) Strain differences in murine ventilatory behavior persist after urethane anesthesia. J Appl Physiol 97: 888-894. doi:10.1152/japplphysiol.01346.2003. PubMed: 15333626. 15333626 10.1152/japplphysiol.01346.2003

[43] Yamauchi M, Dostal J, Kimura H, Strohl KP (2008) Effects of buspirone on posthypoxic ventilatory behavior in the C57BL/6J and A/J mouse strains. J Appl Physiol 105: 518-526. doi:10.1152/japplphysiol.00069.2008. PubMed: 18511527. 18511527 10.1152/japplphysiol.00069.2008 PMC2519940

[44] Song Z, Harris KA, Thach BT (2009) Laryngeal constriction during hypoxic gasping and its role in improving autoresuscitation in two mouse strains. J Appl Physiol 106: 1223-1226. doi:10.1152/japplphysiol.91192.2008. PubMed: 19164773. 19164773 10.1152/japplphysiol.91192.2008 PMC2698640

[45] Chen XJ, Kovacevic N, Lobaugh NJ, Sled JG, Henkelman RM et al. (2006) Neuroanatomical differences between mouse strains as shown by high-resolution 3D MRI. NeuroImage 29: 99-105. doi:10.1016/j.neuroimage.2005.07.008. PubMed: 16084741. 16084741 10.1016/j.neuroimage.2005.07.008

[46] Navarro M, Leza JC, Lizasoain I, Lorenzo P (1991) Influence of psychogenetics in opiate tolerance and abstinence in mice. Gen Pharmacol 22: 713-716. doi:10.1016/0306-3623(91)90084-J. PubMed: 1936907. 1936907 10.1016/0306-3623(91)90084-j

[47] Suaudeau C, Bousselmame R, Costentin J (1992) A different balance in the sensitivity of D1 and D2 dopamine receptors accounts for differences in apomorphine-induced hypothermic effects in Swiss and C3H mice. Neuropharmacology 31: 1115-1119. doi:10.1016/0028-3908(92)90007-C. PubMed: 1475020. 1475020 10.1016/0028-3908(92)90007-c

[48] Ennaceur A, Michalikova S, van Rensburg R, Chazot PL (2006) Models of anxiety: responses of mice to novelty and open spaces in a 3D maze. Behav Brain Res 174: 9-38. doi:10.1016/j.bbr.2006.07.001. PubMed: 16919819. 16919819 10.1016/j.bbr.2006.07.001

[49] Fraigne JJ, Orem JM (2011) Phasic motor activity of respiratory and non-respiratory muscles in REM sleep. Sleep 34: 425-434. PubMed: 21461320. 21461320 10.1093/sleep/34.4.425 PMC3065252

[50] Real C, Seif I, Adrien J, Escourrou P (2009) Ondansetron and fluoxetine reduce sleep apnea in mice lacking monoamine oxidase A. Respir Physiol Neurobiol 168: 230-238. doi:10.1016/j.resp.2009.07.003. PubMed: 19615472. 19615472 10.1016/j.resp.2009.07.003

[51] Megirian D, Pollard MJ, Sherrey JH (1987) The labile respiratory activity of ribcage muscles of the rat during sleep. J Physiol 389: 99-110. PubMed: 3119821. 3119821 10.1113/jphysiol.1987.sp016648 PMC1192072

[52] Krieger J (2005) Respiratory Physiology: breathing in normal subjects. Principles and practice of sleep medicine. 4th ed. Philadelphia, PA: Elsevier/Saunders pp. 232-244.

[53] Remmers JE, deGroot WJ, Sauerland EK, Anch AM (1978) Pathogenesis of upper airway occlusion during sleep. J Appl Physiol 44: 931-938. PubMed: 670014. 670014 10.1152/jappl.1978.44.6.931

[54] Sauerland EK, Harper RM (1976) The human tongue during sleep: electromyographic activity of the genioglossus muscle. Exp Neurol 51: 160-170. doi:10.1016/0014-4886(76)90061-3. PubMed: 177304. 177304 10.1016/0014-4886(76)90061-3

[55] Lu JW, Kubin L (2009) Electromyographic activity at the base and tip of the tongue across sleep-wake states in rats. Respir Physiol Neurobiol 167: 307-315. doi:10.1016/j.resp.2009.06.004. PubMed: 19539786. 19539786 10.1016/j.resp.2009.06.004 PMC2717949

[56] Brooks PL, Peever JH (2011) Impaired GABA and glycine transmission triggers cardinal features of rapid eye movement sleep behavior disorder in mice. J Neurosci 31: 7111-7121. doi:10.1523/JNEUROSCI.0347-11.2011. PubMed: 21562273. 21562273 10.1523/JNEUROSCI.0347-11.2011 PMC6703223

[57] Kato T, Masuda Y, Yoshida A, Morimoto T (2011) Masseter EMG activity during sleep and sleep bruxism. Arch Ital Biol 149: 478-491. PubMed: 22205593. 22205593 10.4449/aib.v149i4.1317

